# Postrecruitment Function of Yeast Med6 Protein during the Transcriptional Activation by Mediator Complex

**DOI:** 10.1155/2018/6406372

**Published:** 2018-01-09

**Authors:** Gwang Sik Kim, Young Chul Lee

**Affiliations:** School of Biological Science and Technology, Hormone Research Center, Chonnam National University, Gwangju, Republic of Korea

## Abstract

Med6 protein (Med6p) is a hallmark component of evolutionarily conserved Mediator complexes, and the genuine role of Med6p in Mediator functions remains elusive. For the functional analysis of *Saccharomyces cerevisiae* Med6p (scMed6p), we generated a series of scMed6p mutants harboring a small internal deletion. Genetic analysis of these mutants revealed that three regions (amino acids 33–42 (Δ2), 125–134 (Δ5), and 157–166 (Δ6)) of scMed6p are required for cell viability and are located at highly conserved regions of Med6 homologs. Notably, the Med6p-Δ2 mutant was barely detectable in whole-cell extracts and purified Mediator, suggesting a loss of Mediator association and concurrent rapid degradation. Consistent with this, the recombinant forms of Med6p having these mutations partially (Δ2) restore or fail (Δ5 and Δ6) to restore in vitro transcriptional defects caused by temperature-sensitive *med6* mutation. In an artificial recruitment assay, Mediator containing a LexA-fused wild-type Med6p or Med6p-Δ5 was recruited to the *lexA* operator region with TBP and activated reporter gene expression. However, the recruitment of Mediator containing LexA-Med6p-Δ6 to *lexA* operator region resulted in neither TBP recruitment nor reporter gene expression. This result demonstrates a pivotal role of Med6p in the postrecruitment function of Mediator, which is essential for transcriptional activation by Mediator.

## 1. Introduction

Promoter-specific mRNA synthesis requires a minimal set of proteins comprising Pol II (RNA polymerase II) and associated GTFs (general transcription factors), which is defined as in vitro “basal transcription” [1]. Regulated responses of this minimal transcription system by DNA-binding transcription factors or in vitro transcription from nucleosomal DNA template require additional sets of coregulator proteins involved in chromatin remodeling/modification as well as targeted recruitment of basal transcription machinery to the promoter [2, 3]. In vivo, these transcriptional cofactors indeed participate in the regulated expression of specific genes in response to environmental and physiological cues, which has been demonstrated in a wide range of eukaryotic model organisms.

Mediator complex was first identified from yeast *S. cerevisiae* [4], and Mediator homologs or its related complexes were subsequently found in other eukaryotic species, placing this evolutionarily conserved coregulator as an essential and general player in Pol II transcription [5, 6]. Yeast Mediator is required for diverse aspects of transcriptional regulation, such as activation, repression, stimulation of basal transcription, and TFIIH-dependent phosphorylation of the CTD (C-terminal repeat domain) of the Rpb1 subunit in vitro [2, 4]. The tight association of Mediator with the CTD of Pol II forms Pol II holoenzyme, which is generally required for the transcription of most class II genes in vivo [7]. Genetic and biochemical studies identified 21 polypeptides as bona fide subunits of the core Mediator, including transcriptional regulators previously identified by genetic studies, as well as the novel subunits collectively named Med proteins [8]. In addition, a subset of Srb/Ssn proteins was identified as the distinct kinase module whose association with core Mediator is known to suppress the positive function of Mediator [9, 10].

Molecular and genetic studies have revealed that some Mediator subunits are specifically required for the regulation of a subset of genes, whereas others are necessary for general transcription of Pol II [11]. Consistent with these observations, our biochemical studies have demonstrated that functionally related Mediator subunits physically associate to form two stable subcomplexes, named Rgr1/Med14 and Srb4/Med17 [12]. The Med14 subcomplex was subsequently shown to contain several functional modules, including Gal11/Med15 module (Med2, -3, -15, and -16) and Med9/10 module (Med1, -4, -7, -9, -10, and -21), which suggested a modular structure of Mediator [12, 13]. While the Med14 subcomplex consists of several functional modules presumed to function in receiving diverse signals from activators or repressors, the Med17 subcomplex, composed of Med6p and genetically dominant Srb proteins (Med17, -18, -20, and -22), is thought to functionally interact with the Rpb1 CTD [12]. Based on electron microscopic (EM) images, the modular structure of core Mediator was represented as comprising distinct head, middle, and tail domains [14]. Recent X-ray structures of yeast Mediators suggest that the Med17 subcomplex corresponds to the head module while the Med14 subcomplex contains both tail and middle modules [15–18]. In holoenzyme and preinitiation complex (PIC) models based on refined EM and X-ray structures, both the middle and head modules make multiple contacts with diverse parts of Pol II and GTFs, explaining the molecular mechanism of Mediator-stimulated CTD phosphorylation by TFIIH and resultant enhancement of basal transcription [15, 16, 19].

Med6p is a hallmark component of evolutionarily conserved Mediator complexes and participates in transcriptional activation of many class II genes in *S. cerevisiae* [5, 20]. In metazoans, mutant flies deficient in dMED6 exhibit transcriptional defects in a wide range of developmentally regulated genes, validating the functional conservation and the requirement for Med6p in Pol II transcription in vivo [21]. Intriguingly, *med17* mutation was isolated as a dominant suppressor of *med6* temperature-sensitive (ts) mutation [12], and this genetic interaction was confirmed by an opposite genetic approach [22]. Consistent with functional interaction, a direct physical association between Med6p and Med17p was identified through successful in vitro reconstitution of the Med17 subcomplex [13, 23]. Recently, X-ray structural data have been obtained for the head modules of *S. cerevisiae* and *S. pombe* Mediators, which indicate that the structures of two head modules are well conserved despite the limited sequence similarities between these regions [17, 18]. The conserved Med6p structure revealed a core domain, which comprises a five-stranded antiparallel *β*-sheet, two pairs of *α*-helices flanking a conserved groove, and a flexible C-terminal *α*5 helix [18]. This domain is called “shoulder,” according to its position relative to the head module, and was shown to interact with other subunits within the head module (Med8 and Med17) and the middle module (Med4, Med10, and Med19) [18]. Recently, the C-terminal *α*6 helix of the Med6 subunit was shown to connect to the Med14 subunit of the middle module as one of the tethering molecules of the head module [15, 16]. These results strongly suggest that the Med6p may function as a critical interface between the head and middle modules and relay a regulatory signal from the tail/middle module to the head module/Pol II as proposed [12].

Although extensive structural studies have been made to reveal the action mechanisms of Mediator in Pol II transcription, analyses of each Mediator component to confirm the functions inferred from structural data remain to be completed. In order to enhance our understanding of the key role of *S. cerevisiae* Med6p (scMed6p) in Mediator functions, we generated a series of Med6p mutants harboring small internal deletions (10 amino acids) and analyzed the importance of each region of scMed6p in transcription activation by Mediator in vivo and in vitro. We identified three distinct domains of scMed6p required for its essential function: one N-terminal region (Δ2, amino acids 33–42) might be involved in Mediator association and/or protein stability, and the second (Δ5, amino acids 125–134) and third (Δ6, amino acids 157–166) regions have a role in postrecruitment function, such as signal-relay or conformational changes during transcriptional activation by Mediator.

## 2. Materials and Methods

### 2.1. Construction of med6 Deletions, Mutants, and Other Expression Plasmids

**Figure 1:**
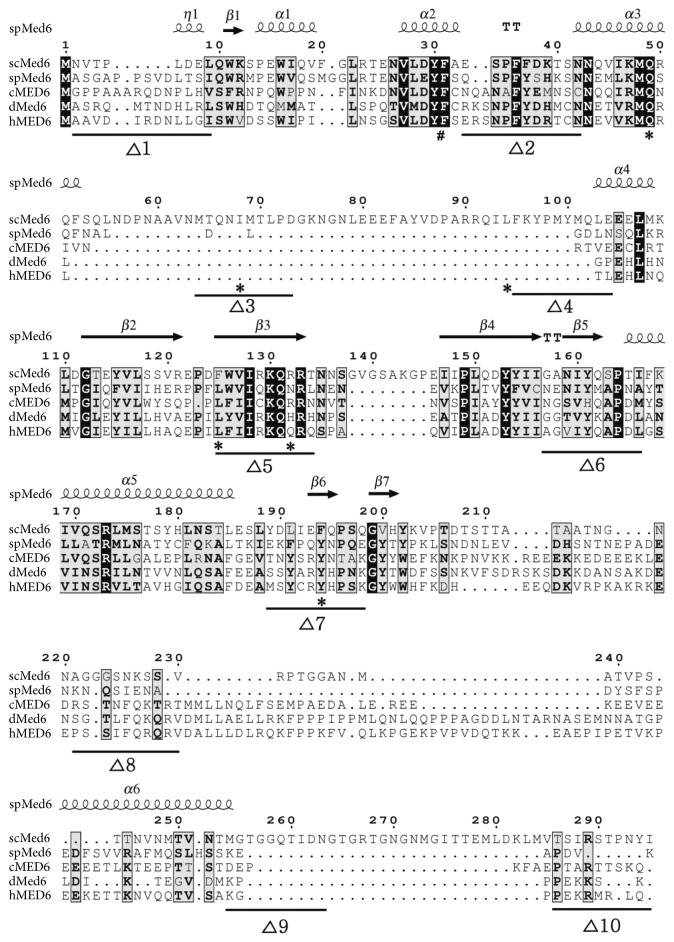
Multiple-sequence alignments of Med6p homologs. Sequences of Med6 proteins from *S. cerevisiae* (scMed6), *S. pombe* (spMed6), *C. elegans* (cMED6), *D. melanogaster* (dMed6), and *H. sapiens* (hMED6) are aligned with the aid of Expresso (Di Tommaso et al. [24]) and presented by ESPript 3.0 program (Robert and Gouet [25]) along with the secondary structure information of spMed6 (pdb code 4H63 and 5N9J). Black and shaded boxes indicate identical and similar amino acids, respectively, and gaps are represented as dots. Ten regions chosen for the internal deletion (10 amino acids each) are also shown as thick lines just below the corresponding amino acid sequences of scMed6p. The mutational sites found in *med6-ts1* and *med6-ts2* alleles are depicted as sharp (#) and asterisk (∗), respectively.

Serial deletion mutants of the *scMED6* gene were constructed by an oligonucleotide-directed mutagenesis method. The *MED6* gene harboring own promoter and terminator regions was obtained from pRS316-MED6 by EcoRI-XbaI digestion and subcloned into the pALTER-1 vector (Promega) for site-directed mutagenesis according to the manufacturer's instructions. A series of mutagenic oligonucleotides were designed to delete 10 amino acids of Med6p spanning the whole *MED6* ORF region (Figure 1). A NarI restriction site was introduced into deletion points within each mutagenic oligonucleotide, which was used for the construction of double deletion mutants (Δ3-4, Δ7-8, and Δ9-10). After the deletions were confirmed by sequencing, mutant med6 genes were subcloned into pRS313 plasmid.

For the copper-inducible expression of Med6p, wild-type and mutant *MED6* genes were subcloned into pC (native form) or pCL (LexA-fused form) vectors using appropriate restriction sites [26]. pGEX-MED6 was made by subcloning the EcoRI-SalI fragment from pGBT-MED6 into pGEX-2TK (Pharmacia). The pGEX-*med6* deletion constructs were made by replacing the indicated region of pGEX-MED6 with the corresponding fragments of mutant constructs in pRS313 by the use of restriction sites. In order to make the *E. coli* expression constructs for 6xHis-tagged Med6p and its deletion derivatives, the EcoRI-ClaI fragments from the pGEX-MED6 and deletion constructs were subcloned into pET vector series.

### 2.2. Growth Complementation Assay

To test the growth complementation by *med6* deletion mutants, yeast strain YCL4 (*MATα*, *ade2*, *his3*, *trp1*, *lys2*, *med6*Δ::*LEU2*, and pRS316-MED6) was used as a host strain for plasmid shuffling. The *med6* deletion mutants in pRS313 were introduced into YCL4 strain and the resulting transformants were tested for their growth on synthetic complete media containing 5-fluoroorotic acid via the plasmid shuffling method.

### 2.3. Protein Purification

Gal4-VP16, GTFs, wild-type, and *med6* ts mutant Pol II holoenzymes were prepared using several chromatographic steps as described previously [20]. Recombinant Med6p and mutant derivatives were expressed in *E. coli* strain BL21(DE) (Novagen) and purified through Ni^+^-NTA columns (Qiagen) under denaturing conditions. Purified proteins were renatured at 4°C through stepwise dialysis according to the protocol described by Thompson et al. [27] and stored at −80°C before use.

### 2.4. In Vitro Transcription

Reconstituted in vitro transcription was performed as described previously [20]. Either wild-type or med6 ts mutant Pol II holoenzymes were preincubated for 10 min at 25°C or 37°C with other supplements containing two DNA templates (pGAL4:G- and pGCN4:G-), GTFs, 0.5 mM ATP, and Gal4-VP16, with or without recombinant Med6p derivatives. After initiation complex formation, the transcription reaction (30 min, 25°C) was initiated by the addition of [*α*-^32^P]-UTP and CTP. The purified reaction products were analyzed on the 7% denaturing PAGE and autoradiography.

### 2.5. LacZ Reporter Assay, GST Pull-Down Analysis, Western Analysis, and Chromatin Immunoprecipitation

For the analysis of reporter gene expression in yeast, yeast strains were grown to the mid-log phase in selective synthetic complete media and *β*-galactosidase activity of cultured cells was measured in triplicate by the permeabilized-cell method [28]. To investigate the expression levels of mutant Med6ps, yeast strain YCL4 was transformed with plasmids encoding LexA-Med6p derivatives (in pCL313) and/or native forms of Med6p derivatives (in pC314). The resulting transformants were cultured in synthetic glucose media containing 0.5 mM CuSO_4_ for 2 h to induce the expression of Med6 proteins. Preparation of yeast whole-cell extracts (WCEs), western analysis, and immunoprecipitation of Mediator were performed according to the protocols described previously [12]. For chromatin immunoprecipitation, L40 yeast strains expressing LexA-Med6p derivatives (in pCL313) were grown in synthetic media containing glucose (Glc) or galactose plus raffinose (Gal + Raf) for 6 h in the presence of 0.5 mM CuSO_4_. Cells were harvested, fixed in 1% formaldehyde solution, and sonicated for the fragmentation of chromatin according to the protocol described by Kuo and Allis (1999). WCEs containing chromatin fragments were immunoprecipitated with the use of appropriate antibodies and subjected to a decrosslinking reaction. Purified DNAs were subjected to PCR (25 cycles) with the use of oligomers specific to promoter regions of *LacZ* reporter or *GAL1-10* genes, respectively.

## 3. Results

### 3.1. Identification of yMed6p Regions Required for Cell Viability

**Figure 2:**
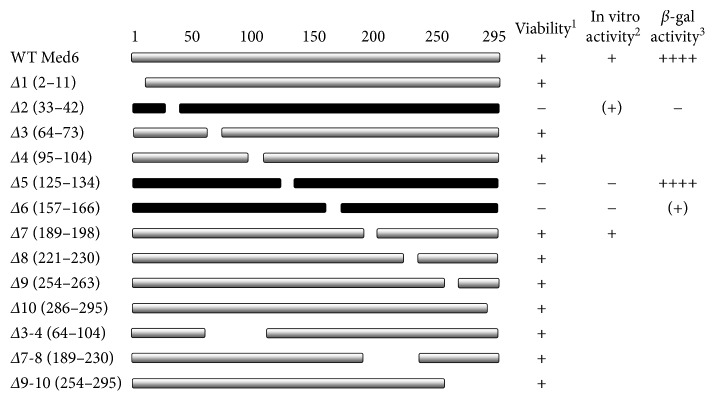
Schematic presentations of Med6p derivatives and summary of their functional defects in vivo and in vitro. Viability^1^ was tested for the ability of deletion mutants to complement the *med6* null mutation via plasmid shuffling as described in Materials and Methods. In vitro activity^2^ represents the capability of each recombinant form of Med6 proteins to rescue the in vitro transcriptional defects of *med6-ts2* holoenzyme based on the quantitative data shown in Figure 4. *β*-galactosidase activity^3^ indicates the expression levels of the reporter gene in the artificial recruitment assay of the indicated Med6p derivatives as shown in Figure 6.

**Figure 3:**
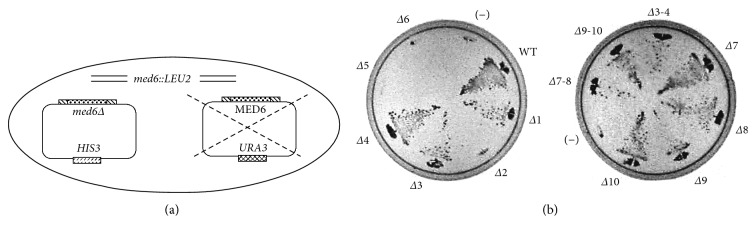
Identification of the regions in Med6p required for cell viability. (a) Schematic depiction of plasmid shuffle technique for the complementation test of mutant *MED6* genes (*med6*Δ). (b) The growth complementation test of the *med6* deletion mutants. Wild-type (WT) *MED6* and indicated *med6* deletion mutants in the pRS313 vector were introduced into the host strain YCL4 (*med6*Δ::*LEU2* and pRS316-MED6) for plasmid shuffling. The resulting transformants were grown for 4 days at 30°C on synthetic complete media containing 5-fluoroorotic acid to remove the pRS316-MED6. (–): pRS313 vector only.

For the unbiased and systematic analysis of yMed6p, a series of Med6p mutants harboring a small internal deletion was made. We designed 10 amino acid deletions throughout the entire Med6p for approximately every 30 amino acid interval and introduced these into *MED6* genes harboring own promoter and terminator regions (Figures 1 and 2). The mutant *med6* constructs were then transformed into host strain YCL4 for plasmid shuffling and tested for supporting cell viability upon the removal of wild-type *MED6* gene in pRS316 by 5-fluoroorotic acid treatment (Figure 3). Despite the considerable size for deletions (10 amino acids), seven out of ten deletion mutations (Δ1, Δ3, Δ4, Δ7, Δ8, Δ9, and Δ10) had no obvious effect on the viability of yeast cells (Figures 2 and 3). Notably, the *med6* deletion mutants containing Δ2 (residues 33–42), Δ5 (residues 125–134), or Δ6 (residues 157–166) were unable to support the cell growth.

The multiple-sequence alignment of Med6p homologs using the Expresso program indicated a high degree of conservation between yeast and metazoan Med6 proteins (Figure 1). However, scMed6p has distinct features from other Med6 homologs in four regions: two internal and one C-terminal spacer regions (50, 8, and 30 amino acids long, resp.) that are exclusively found in scMed6p and one variable region among Med6 homologs (amino acids 230–240 in scMed6p). According to alignment data, three regions (Δ2, Δ5, and Δ6), identified as essential for *MED6* function(s), closely matched the highly conserved regions of Med6 homologs (Figure 1).

To confirm the functional regions of scMed6p, larger deletion mutants were made and used in a complementation test. First, we constructed a Med6p mutant having the combined deletion of Δ3 and Δ4 (Δ3-4), resulting in the removal of the first region specific for scMed6p. Interestingly, this mutant could fully complement the *MED6* function, indicating that this region is dispensable for essential function (Figures 2 and 3). In addition, one-third of the C-terminal domain of scMed6p, corresponding to the most divergent region of Med6p, also proved to be unnecessary for *MED6* function, since two mutants (Δ7-8 and Δ9-10) spanning this region had no obvious effect on cell survival (Figures 2 and 3). This assumption was confirmed by the fact that scMed6p comprising only residues 1–210 could support cell viability (see Supplementary Figure 1).

Taken together, the functional domains of yMed6p essential for cell viability were mapped to the highly conserved regions of Med6p, whereas the scMed6p-specific region and the divergent region of Med6ps were not required for essential function(s) of scMed6p.

### 3.2. Phenotypes or Transcriptional Defects of the Viable Med6 Mutants

Although the deletion analysis indicated that substantial parts of scMed6p were dispensable for cell viability, a significant level of sequence conservation is apparent at these regions, including Δ1 and Δ7 regions. Thus, we investigated whether the viable *med6* deletion mutants showed any growth defects or transcriptional defects in vivo, such as ts lethality or limited carbon source utilization, as observed in *med6* ts mutants [12]. All of the viable mutant strains had no observable ts phenotype and utilized galactose or raffinose at 30°C as efficiently as the wild-type did (Supplementary Figure 2A).

Based on the fact that *MED6* is essential for galactose-induced *GAL1* gene activation [20], *β*-galactosidase activity of the *GAL1*_*UAS*_-TATA-*LacZ* reporter was measured for the viable mutants under induction conditions. None of the viable *med6* deletion mutants were defective in transcriptional activation of the *GAL1* promoter (Supplementary Figure 2B). These results strongly suggest that the pivotal function(s) of scMed6p resides in the evolutionarily conserved regions.

### 3.3. In Vitro Transcriptional Activity of Mutant Med6 Proteins

**Figure 4:**
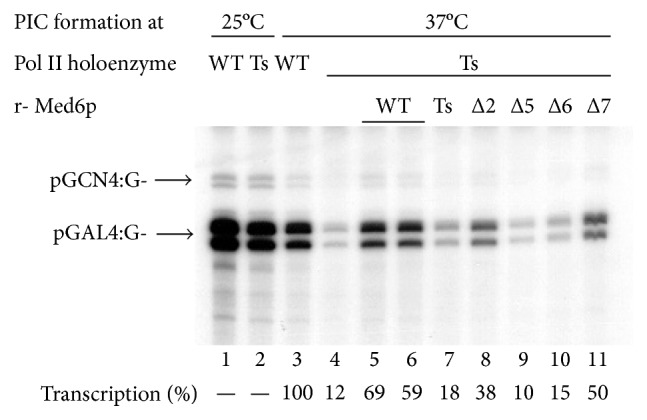
Three lethal Med6p mutants also show transcriptional defects in a reconstituted in vitro system. Either wild-type (WT) or *med6 ts-2* mutant (Ts) Pol II holoenzyme (700 ng each) was preincubated for 10 min at 25°C or 37°C with other supplements containing two DNA templates, GTFs, 0.5 mM ATP, and Gal4-VP16 (30 ng), with or without the indicated recombinant Med6p (80 ng each). After PIC formation, the transcription reaction (30 min, 25°C) was initiated by the addition of [*α*-^32^P]-UTP and CTP. The mRNA products were purified and resolved on 7% denaturing polyacrylamide gel, followed by autoradiogram. Transcription (%) was set to 100% as to the fold activation (signal from GAL4 template divided by signal from GCN4 template) shown by wild-type holoenzyme upon PIC formation at 37°C. The data quantitation was performed with the use of Personal Molecular Imager FX system and associated program (Bio-Rad).

Next, we investigated whether scMed6p regions essential for viability are also required for Med6p-dependent transcriptional activation in vitro. For this, we purified recombinant Med6p having *ts-2* mutation or internal deletion mutations and examined their capabilities in restoring the transcriptional defects of Pol II holoenzyme caused by *med6 ts-2* mutation [20]. When transcriptional PIC was formed at 25°C, both wild-type and *med6-ts2* Pol II holoenzymes showed 23- and 21-fold activation of *GAL4* enhancer-containing template (GAL4:G-) by Gal4-VP16 over basal transcription from GCN4:G-template, respectively (Figure 4, lanes 1 and 2). However, PIC formation at 37°C specifically impaired the transcription activity of *med6-ts2* holoenzyme in that the mutant holoenzyme gave only a 2.5-fold activation in comparison to the 21-fold activation by wild-type holoenzyme (Figure 4, lanes 3 and 4). This temperature-dependent transcriptional defect of mutant holoenzyme was recovered by the addition of the recombinant form of wild-type Med6p, but not Med6-ts2 protein, prior to the PIC formation at 37°C (lanes 5–7). Next, we examined whether each deletion mutant of Med6p (-Δ2, -Δ5, -Δ6, and -Δ7) had the ability to restore the transcriptional defect of *med6-ts2* holoenzyme. The Med6p-Δ7, which showed no apparent defect in vivo, did rescue the activation defect of the heat-inactivated *med6-ts2* holoenzyme (lane 11). However, Med6p-Δ5 and -Δ6 mutants were not able to complement this defect (lanes 9 and 10), in good agreement with their in vivo phenotypes. Notably, Med6p-Δ2, which did not support cell viability, could partially restore the transcriptional defect of *med6-ts2* holoenzyme to half that of the wild-type (lanes 5, 6, and 8; see Discussion). These results indicate that the Med6p regions which are essential for in vivo function (cell viability) are also required for activation function of Med6p in vitro.

### 3.4. Med6p-Δ2 Has a Defect in Association with Mediator

**Figure 5:**
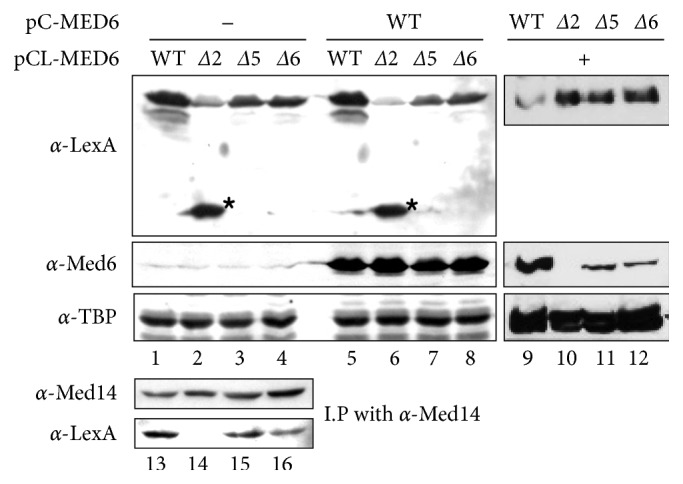
The deficiency of Med6p-Δ2 in WCEs and purified Mediator fraction. (lanes 1–12) WCEs were prepared from YCL4 yeast strains expressing the indicated LexA-Med6p derivatives (pCL-MED6) along with (+) or without (−) coexpression of the native forms of Med6p derivatives (pC-MED6) by copper induction for 2 h. Samples were resolved on an 8% denaturing polyacrylamide gel, and expression levels of LexA-fused and native forms of Med6p derivatives were examined by western analysis using anti-LexA antibody and anti-Med6 antibody, respectively. Western analysis for TBP was done for loading control. The degradation product of LexA-Med6p-Δ2 is indicated by an asterisk. (lanes 13–16) WCEs were prepared from the indicated yeast strains and subjected to DEAE column fractionation to enrich the Pol II holoenzyme. Pol II holoenzyme in each sample was immunopurified with beads-coupled anti-Med14 antibody and subjected to western analysis to measure the amounts of LexA-Med6p derivatives (*α*-LexA) contained in the immunopurified Mediator fraction (*α*-Med14).

**Figure 6:**
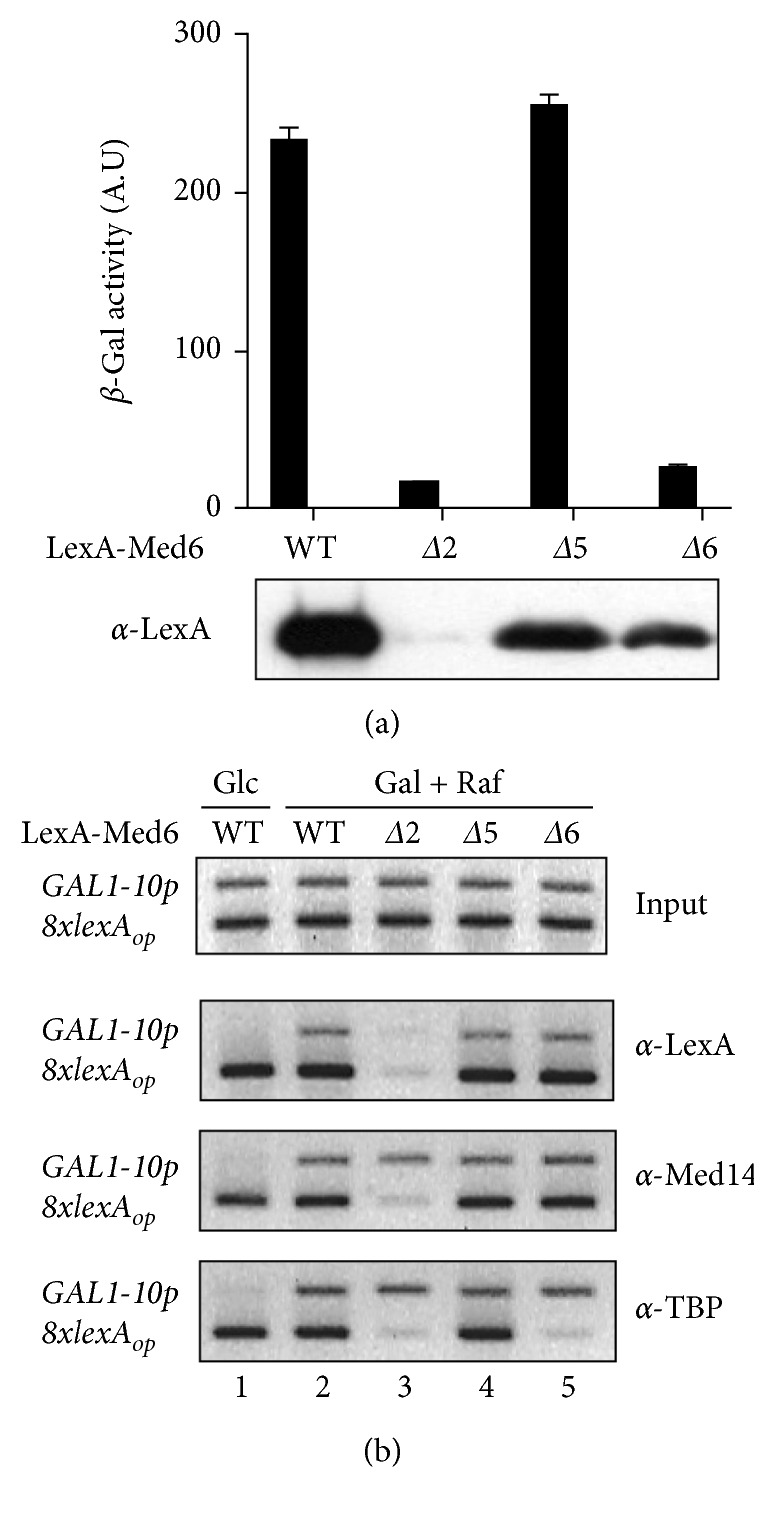
Artificial recruitment assay and chromatin immunoprecipitation analysis of Med6 mutants. (a) Reporter gene activation by targeted recruitment of Mediator via LexA-fused Med6p derivatives. Yeast strain L40 harboring pCL313-Med6 derivatives were cultured in synthetic glucose media containing 0.5 mM copper. The *β*-galactosidase activity of chromosomal 8x*lexA*_*op*_-*LacZ* reporter gene in cultured cells was measured in triplicate. (b) Chromatin immunoprecipitation for recruitment of Mediator (*α*-Med14), LexA-Med6ps (*α*-LexA), and TBP (*α*-TBP) to promoter regions of chromosomal reporter (8x*lexA*_*op*_) and *GAL1-10* (*GAL1-10p*) genes. L40 strains expressing indicated pCL313-Med6 derivatives were grown in synthetic media containing glucose (Glc) or galactose plus raffinose (Gal + Raf) for 6 h in the presence of copper. Chromatin fragments were prepared from WCEs and immunoprecipitated with the use of the indicated antibodies. DNAs were purified from immunoprecipitates and subjected to PCR (25 cycles) with the use of oligomers specific to promoter regions of reporter gene (PCR product, 200 bp) or *GAL1-10* gene (PCR product, 370 bp), respectively. The amount of DNA used for INPUT PCR corresponds to 1% of the immunoprecipitated DNA samples used in PCR.

In our previous reports, the Pol II holoenzyme prepared from *med6-ts* mutants is deficient in the *med6-ts* protein but the free form of *med6-ts* protein was not detected during column purifications, possibly due to its rapid degradation [12, 20]. This result suggests that all the Med6p mutants detected in WCEs appear to be associated with Mediator in vivo. Since the malfunction of three Med6p mutants (Δ2, Δ5, and Δ6) could be reasoned from disabled interaction with Mediator, we investigated the expression levels of LexA-fused forms of Med6p mutants in WCEs in the presence of wild-type Med6p expressed by own promoter. Upon copper induction, the expression level of LexA-Med6p-Δ5 and -Δ6 mutants was about half that of the wild-type, whereas the Δ2 mutant was nearly absent in the prepared WCEs (Figure 5, lanes 1–4; Figure 6). The additional copper induction of the native form of wild-type Med6p resulted in a similar pattern, indicating that overexpression of wild-type Med6p does not affect the expression pattern of these mutants (Figure 5, lanes 5–8). Notably, LexA-Med6p-Δ2 showed a degradation pattern as indicated by the detection of only LexA-sized protein, demonstrating the absence of Med6p-Δ2 in the cell lysate was not due to gene expression (lanes 2 and 6). Consistent with this, LexA-Med6-Δ2p was totally absent in immunopurified Mediator as compared to wild-type and other mutant Med6ps (lanes 13–16). This observation was reproduced when WCEs were prepared from yeast strains expressing LexA-fused wild-type Med6p and native forms of Med6p mutants (Figure 5, lanes 9–12). In this case, we could clearly observe the reciprocal expression pattern between LexA-fused and native forms of Med6ps, indicating their competitive incorporation into Mediator. From these results, we concluded that Med6p-Δ2 has a defect in Mediator association and the Δ2 region (amino acids 33–42) of scMed6p might be involved in the direct interaction with other Mediator subunit(s).

### 3.5. Artificial Recruitment Assay with the Functionally Defective Med6p Mutants

The artificial recruitment of Mediator to a promoter region via Mediator subunit fused with DNA-binding domain resulted in activator-bypass activation of target promoter. We successfully performed an artificial recruitment assay by using Mediator containing LexA-Med6p [29]. Thus, we used LexA-fused Med6p mutants for the artificial recruitment assay to activate the chromosomal 8x*lexA*_*op*_-*LacZ* reporter gene. As expected from the expression data, LexA-Med6p-Δ2 was completely inactive for reporter gene activation (Figure 6). Intriguingly, LexA-Med6p-Δ5 had comparable transcriptional activity to that of wild-type Med6p, suggesting its transcriptional defect can be reversed by artificial recruitment via an unknown mechanism (see Discussion). In the case of the Med6p-Δ6 mutant, its artificial recruitment had no effect on reporter gene expression (Figure 6).

Next, we performed chromatin immunoprecipitation analysis to examine in vivo association of LexA-Med6p derivatives, Med14 (for Mediator), and TBP (for transcription initiation) with a target promoter region (8x*lexA*_*op*_). As shown in Figure 6, Mediator containing LexA-fused Med6p-Δ5 or -Δ6 was recruited to the 8x*lexA*_*op*_ region with an efficiency similar to that of wild-type (irrespective of growth conditions). However, the association of TBP with the promoter region was observed only in the strains expressing LexA-fused wild-type Med6p or Med6p-Δ6, in agreement with reporter gene expression (Figure 6).

Additionally, we examined the recruitment of Med6p mutants to an endogenous *GAL1-10* promoter region under either repression (glucose) or induction conditions (galactose plus raffinose) to monitor the effect of *med6* mutations on the recruitment of Mediator to a natural promoter. LexA-fused Med6p-Δ5 and -Δ6 were recruited to the *GAL1-10* promoter as well as wild-type Med6p upon galactose induction, indicating these mutations did not impair Mediator recruitment in the endogenous promoter (Figure 6). In other words, in vivo defects of Med6p-Δ5 and -Δ6 did not result from a problem in Mediator-recruitment step. Promoter association of TBP (*α*-TBP) was also seen in all cases, as predicted by the action of native form of Mediator containing endogenous wild-type Med6p for the activation of *GAL1-10* promoter (Figure 6). All these results directly demonstrate a pivotal role of Med6p in postrecruitment function (i.e., signal-relay or conformational changes) of Mediator during transcriptional activation, even though the Δ5 and Δ6 regions of Med6p might have distinct roles as suggested by the different responses in the artificial recruitment assay (Figure 6; see Discussion).

## 4. Discussion

In this report, we dissected the functional domains of yeast Med6p on the basis of its requirement for cell viability and transcriptional activation via molecular genetics and biochemical approaches. The functional domains of scMed6p (Δ2, Δ5, and Δ6 regions) identified by complementation assays were mapped to the highly conserved regions of Med6ps, whereas yeast-specific (spacer region) and mostly divergent regions (one third of C-terminus) of scMed6p were not required for its essential function(s) (Figures 1 and 2). Three lethal deletion mutants had defects in transcriptional activation in a reconstituted in vitro system although only the Gal4-VP16 activator was tested in our in vitro transcription (Figure 4). These results again clearly demonstrate that the major and essential function of Med6p is focused on transcriptional activation.

One interesting point is that some residual activity to support in vitro transcription was retained in Med6p-Δ2, showing a discrepancy with its in vivo defect. Although we cannot explain clearly this phenomenon, we surmise that this partial activity might have resulted from an in vitro dosage effect. If Med6p-Δ2 has a normal activation function but has a defect in Mediator association, Med6p-Δ2 could provide in vitro activation function to some extent via a dosage effect when an excess amount of Med6p-Δ2 is added (which is not applicable in vivo).

In contrast to Med6p-Δ5 and -Δ6 mutants, the Med6p-Δ2 protein was barely detectable in WCEs and was even absent in enriched Pol II-Mediator fraction. As mentioned above, the comparable proportions of *med6-ts* proteins detected in WCEs seem to be associated with Mediator in vivo. The observed deficiency and the degradation pattern of Med6p-Δ2 in WCEs suggest that the Δ2 region of scMed6p might be involved in the association with other Mediator subunit(s) (Figure 5). Based on recent X-ray structure of *S. pombe* Mediator in PIC, the C-terminal residues of *α*3 helix of spMed6p are shown to directly interact with the Med10/Nut2 subunit to form a head-middle module interface [16]. Since the Δ2 region of scMed6p contains N-terminal residues of *α*3 helix, its deletion might disrupt Med6-Med10 interaction by affecting *α*3 helix structure (Figure 1). In this scenario, the dissociated form of the Δ2 mutant seems to be rapidly removed via specific degradation pathway probably due to its instability. Notably, one (Q49L) of three critical mutations in the *med6-ts2* allele is found in the *α*3 helix of scMed6p, suggesting the molecular basis of the deficiency of *med6-ts2* protein in WCEs and purified Mediator [20]. The other two mutations (I68L and L94P) were mapped to the Δ3 and Δ4 regions, respectively, which correspond to the nonessential spacer region of scMed6p (Figure 1).

Previous biochemical analyses and artificial recruitment experiments using mutant Pol II holoenzymes suggested that facilitated recruitment of a holoenzyme to a promoter is necessary but not sufficient for transcriptional activation and that an unknown biochemical activity is required for transcriptional activation (postrecruitment step) [29]. Consistent with this, the structural data suggested that Med6p may function as a critical interface between the head and middle modules, and relay a regulatory signal from the tail/middle module to the head module/Pol II [15–17]. This signal relay seems to be achieved via multiple interactions of Med6p with other subunits: Med14 in the tail/middle module; Med4, Med10, and Med19 in the middle module; and Med8 and Med17 in the head module [15, 16].

Our artificial recruitment assay revealed that the transcriptional defects of Med6p-Δ5 and -Δ6 in vivo did not result from a problem in the Mediator-recruitment step (Figure 6), which is mainly achieved by an activator-tail module interaction. In this regard, the defects of Med6p-Δ5 and -Δ6 mutants in activated transcription might reflect their impairments in activation signal relay and inabilities to accomplish intermediary functions that are essential for cell viability. Intriguingly, the transcriptional defects of Med6p-Δ5 seem to be rescued by an artificial recruitment (Figure 6). This activator-bypass activation was observed in an artificial recruitment experiment using a holoenzyme devoid of the tail module, which is defective in the Mediator-recruitment step [29]. From this, we proposed that in vivo defect of Med6p-Δ5 could be the result of a flaw in the reception of upstream activation signal from the tail/middle module, which can be rescued by an artificial recruitment. According to the structural data, Δ5 region, corresponding to *β*3 sheet of scMed6p (Figure 1), is critical to form a core domain of Med6p but is not documented to interact directly with other Mediator subunit(s) [18]. In contrast to the Δ5 mutant, the Med6p-Δ6 protein was completely inactive in the transcriptional activation when artificially recruited to target the reporter (Figure 6). The Δ6 region of scMed6p spans *β*5 sheet and N-terminal part of *α*5 helix based on X-ray structure [18]. Since *α*5 helix makes critical contacts between Med8 and Med17 subunits within the head module, the deletion of Δ6 region might cause the defective interactions of Med6p with these subunits. According to this scenario, the Med6p-Δ6 protein cannot relay the activation signal from the tail/middle module to the head module, which is not rescued by an artificial recruitment as previously shown by Med18/Srb5-deficient holoenzyme [29].

Collectively, all these results consistently support a notion that Med6p has a pivotal role in the postrecruitment function of Mediator during transcriptional activation, which is conceptually accepted as the conformational changes, isomerization, or modulation of Pol II activity. In addition, our results also suggest there might be functional differentiation between the Δ5 and Δ6 regions (involved in the relay of *upstream* versus *downstream* signal). Future studies on the detailed functions of these essential domains of Med6p should be helpful for the understanding of action mechanisms of Mediator with regard to its structural and functional relationship.

## 5. Conclusion

Through the systematic dissection of functional domains of scMed6p, we identified three distinct domains of scMed6p required for its essential function: one N-terminal region (Δ2, amino acids 33–42) might be involved in Mediator association and/or protein stability, and the second (Δ5, amino acids 125–134) and third (Δ6, amino acids 157–166) regions have a role in postrecruitment activity of Mediator complex, such as signal-relay or conformational changes. This study proved the previous notion that Med6p is involved in the postrecruitment function of Mediator complex, which is required for gene activation by Mediator.

## Abbreviations

CTD: C-terminal repeat domain
GTFs: General transcription factors
Pol II: RNA polymerase II
ts: Temperature-sensitive
PIC: Preinitiation complex
TBP: TATA-binding protein
WCEs: Whole-cell extracts.

## Supplementary Materials

Supplementary Materials Supplementary Figure 1: The growth complementation test of Med6p comprising residues 1–210. Supplementary Figure 2: The viable Med6 mutants showed no detectable phenotypes or transcriptional defects.
