# Supplementary material for: Postrecruitment Function of Yeast Med6 Protein during the Transcriptional Activation by Mediator Complex
Source: Biochem Res Int. 2018 Jan 9;2018:6406372. doi: 10.1155/2018/6406372 (PMC5818915; doi:10.1155/2018/6406372)
Supplement: Supplementary Materials — Supplementary Figure 1: The growth complementation test of Med6p comprising residues 1–210. Supplementary Figure 2: The viable Med6 mutants showed no detectable phenotypes or transcriptional defects. [file 6406372.f1.pdf]

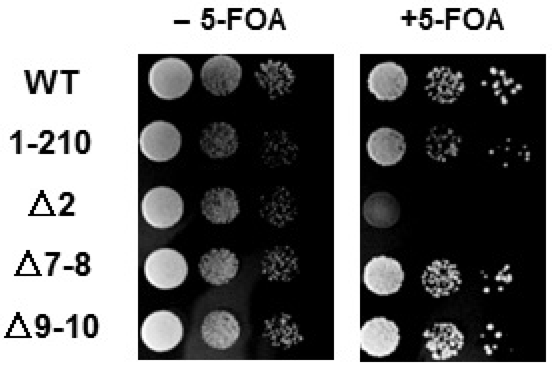

Supplementary Figure 1. The growth complementation test of Med6p comprising residues 1-210 via spot assay.

Wild-type (WT) MED6 and indicated *med6* deletion mutants in pRS313 were introduced into strain YCL4 and the transformants were tested for their growth on synthetic complete media with (+) or without (–) 5-fluoroorotic acid (5-FOA). The same amount of cell solution was spotted on the indicated agar plates with 10-fold serial dilution and incubated for 3-4 days at 30 °C.

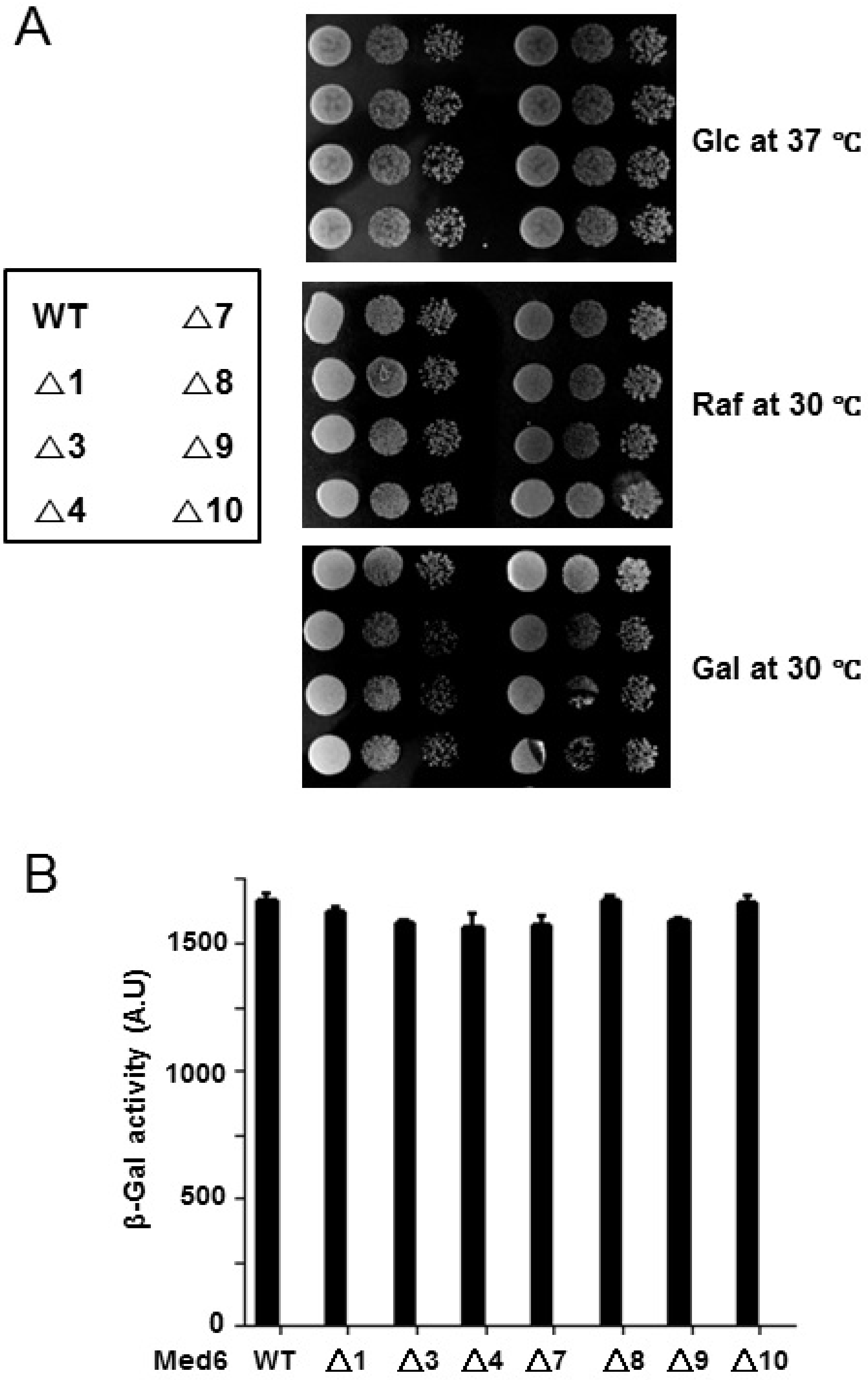

Supplementary Figure 2. The viable *med6* deletion mutants had no observable ts phenotype and utilized galactose or raffinose as efficiently as the wild-type.

(A) Spot assay. Wild-type (WT) MED6 and indicated *med6* deletion mutants were tested for their growth on synthetic complete media containing glucose (Glc), raffinose (Raf), or galactose (Gal) at 37 °C or 30 °C. The same amount of cell solution was spotted on the agar plates with 10-fold serial dilution and incubated for 3-4 days at the indicated temperature.

(B) Reporter assay. The viable *med6* deletion mutants had no defect in galactose-induced *GAL1* gene activation. The $GAL1_{UAS}$-TATA-*LacZ* reporter plasmid was introduced in the indicated MED6 strains and cultured in raffinose media to O.D$_{600}$=0.1. The galactose is added to the culture solution (final 2%) and incubated at 30 °C for 4 h. The β-galactosidase activity of the cultured cell was measured as described in materials and methods. A.U: arbitrary unit.
